# Endurance

**Published:** 1869-11

**Authors:** 


### ENDURANCE.

The commonest street-sweeper, rail-splitter, or wood-cutter has more practical good sense than was possessed by the combined crew of the college-bred Harvard boat-racers. The man who is accustomed to fell the giant trees of the forest takes his first strokes with great deliberation. The experienced horseback traveller begins the day's journey in a deliberate walk, and as the animal warms up it is put to a greater speed. It is the wisdom of public speakers to begin an address in a low, slow, measured tone, bringing the lungs and vocal organs into gradual play; the man who does this may speak an hour with ease; while if he begins on a high key he will begin to cough, and fail in the first five minutes.

The probabilities are that the Harvard crew would have made a more successful row if they had begun more leisurely, and then as they warmed up they could have given a more efficient pull.

---

"Would you," says De Quincey, "desire at this day to read our noble language in its native beauty, picturesque from its idiomatic propriety, racy in its phraseology, delicate yet sinewy in its composition, seal the mail-bags and break open all the letters in female handwriting." The class of women thus alluded to as the true and best depositaries of the old mother idiom are those who, having leisure, have also intelligence, cultivation and thoughtfulness.

One ton of good coal gives off from nine to ten thousand cubic feet of gas. Every cubic foot of gas burned requires ten cubic feet of air for its perfect combustion.

It has been computed that 3,000 cubic miles of water are evaporated every year from the several surfaces of seas, lakes, rivers and wet lands of the globe. The same quantity necessarily falls again in the form of rain and dew.

